# GPS Satellite Orbit Prediction at User End for Real-Time PPP System

**DOI:** 10.3390/s17091981

**Published:** 2017-08-30

**Authors:** Hongzhou Yang, Yang Gao

**Affiliations:** 1 Profound Positioning Inc., Calgary, AB T2P 3G3, Canada; 2 Department of Geomatics, University of Calgary, Calgary, AB T2N 1N4, Canada

**Keywords:** real-time PPP, orbit prediction, initial parameters, numerical integration, user end

## Abstract

This paper proposed the high-precision satellite orbit prediction process at the user end for the real-time precise point positioning (PPP) system. Firstly, the structure of a new real-time PPP system will be briefly introduced in the paper. Then, the generation of satellite initial parameters (IP) at the sever end will be discussed, which includes the satellite position, velocity, and the solar radiation pressure (SRP) parameters for each satellite. After that, the method for orbit prediction at the user end, with dynamic models including the Earth’s gravitational force, lunar gravitational force, solar gravitational force, and the SRP, are presented. For numerical integration, both the single-step Runge–Kutta and multi-step Adams–Bashforth–Moulton integrator methods are implemented. Then, the comparison between the predicted orbit and the international global navigation satellite system (GNSS) service (IGS) final products are carried out. The results show that the prediction accuracy can be maintained for several hours, and the average prediction error of the 31 satellites are 0.031, 0.032, and 0.033 m for the radial, along-track and cross-track directions over 12 h, respectively. Finally, the PPP in both static and kinematic modes are carried out to verify the accuracy of the predicted satellite orbit. The average root mean square error (RMSE) for the static PPP of the 32 globally distributed IGS stations are 0.012, 0.015, and 0.021 m for the north, east, and vertical directions, respectively; while the RMSE of the kinematic PPP with the predicted orbit are 0.031, 0.069, and 0.167 m in the north, east and vertical directions, respectively.

## 1. Introduction

PPP is the state space solution to derive centimeter-level positioning accuracy using a single receiver and IGS precise clock and orbit products [1]. Since it doesn’t need simultaneous measurements from a base receiver station nearby, the hardware requirements will be largely reduced compared to the conventional double difference relative positioning. 

A crucial aspect for real-time PPP [2] is the availability of precise real-time satellite orbit and clock products. In recognition of this demand from the user community, the IGS formed a real-time working group in 2001 to develop technologies, standards and infrastructure for real-time service (RTS). Currently, the IGS RTS are broadcasted to the real-time users through the Internet [3]. There are also a number of companies to provide precise orbit and clock corrections as commercial services to support real-time PPP such as Trimble, Fugro, NavCom, NovAtel, and Hemisphere GNSS [4,5]. 

However, current real-time PPP system using IGS RTS and other commercial services require a continuous connectivity for PPP users to continuously receive precise orbit and clock corrections at a high update rate. A high data rate of corrections will not only mean a large amount of corrections data to transmit to PPP users, but also a requirement on a continuous connection to the correction data server by the PPP users. It also equivalently increases PPP users’ dependence on the correction data in the field, since any loss of correction data will degrade the obtainable positioning accuracy before a network connection is recovered. To ensure the reception of corrections by the users, commercial services usually deploy more L-band communication satellites as backup communication channels to ensure that two communication satellites are always available and in view for users in their service area. However, this still cannot address the problem when positioning in areas with signal blockages/attenuations or poor network signal strength, which is not uncommon in real-time applications. Data communication cost will also be a concern for many applications, particularly for precision applications with mobile devices. Those applications are expected to be the major user community for PPP techniques in the future if the cost concern can be addressed. 

**Figure 1:**
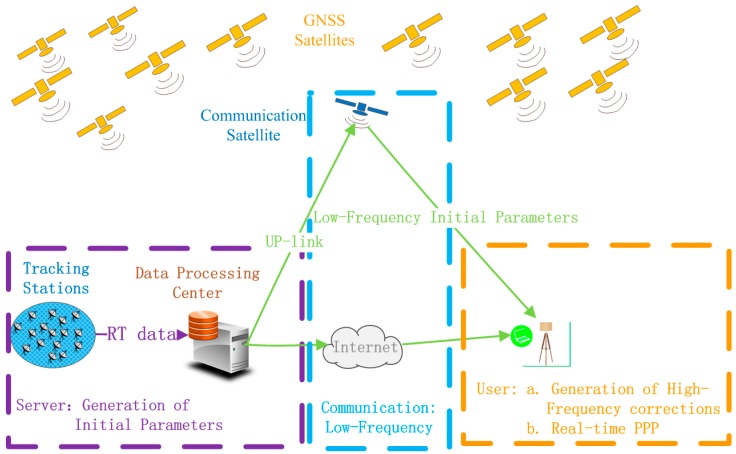
System architecture of the new real-time PPP system.

In order to address those challenges, the concept of a new real-time PPP system has been proposed in Gao et al. [6]. The system architecture of the new real-time system is provided in Figure 1. As we can see from Figure 1, the new system consists of three parts: the server end, the communication end, and the user end. First, it requires the deployment of globally distributed reference stations to contribute real-time network GNSS raw observation data to the processing center at the server end to generate real-time orbit and clock IP. Second, IP generated at the server end need to broadcast to users via communication networks, such as communication satellites or the Internet. Third, PPP users apply initial parameters to generate corrections for orbit and clock error mitigation and precise position determination. The most significant advantage of the new system over existing real-time PPP systems is that the initial parameters broadcast to users can be at a much lower update rate than the precise orbit and clock corrections broadcast by current real-time PPP systems. The high update rate orbit and clock corrections in the new real-time PPP system are to be generated based on the low update rate initial parameters by the user system, instead of receiving from the server system via high-connectivity communication systems. As a result, the concerns mentioned earlier can be effectively addressed. 

This paper will focus on the satellite orbit part. The satellite orbit IP will be generated at the server end with a relatively low update rate, and the contents include the satellite coordinates, satellite velocities, and satellite SRP parameters, which are shown in the following Table 1. The number of the IP that need to be broadcasted each time is 11*n* or 15*n*, which depends on the selection of the satellite SRP model, and *n* is the number of satellites. The IP generated at the server end will be transmitted after the request from the user end through the Internet.

## 2. High Precision Orbit Prediction by Numerical Integration 

### 2.1. Generation of Satellite Orbit Initial Parameters at Server End 

One key point for generating the low update rate orbit IP is the calculation of the forces on navigation satellites or the dynamic models that consist of a set of force models, which can be expressed as the acceleration components:

(1) $$\vec{a}=\sum_{}{\vec{a}}_{i}$$

where the acceleration components include the Earth’s gravitational acceleration, the gravitational acceleration of the Moon and the Sun, the SRP acceleration, and any other independent accelerations acting on the satellite that can be modeled [7]. For the Earth’s gravitational acceleration, the Earth gravitational model (EGM2008) [8] geopotential up to degree 12 and order 12 with the corrections (solid tide, polar tide, and ocean tide) recommended in international Earth rotation and reference systems service (IERS) conventions 2010 [9] will be used. When calculating the gravitational acceleration of the Moon and Sun, the coordinates of the Moon and Sun should be firstly known, which can be obtained from the Jet Propulsion Laboratory (JPL) development ephemeris (DE) series ephemeris [10]. The SRP is one of the crucial parameters in orbit prediction, and in this study, the empirical CODE orbit model (ECOM) of the Center for Orbit Determination in Europe (CODE) is applied with an optional five or nine parameters [11]. As we can see from Table 2, the Earth’s gravitational acceleration has the largest effect on satellite motion, and the magnitude can be several 1 × 10^−1^ m/s^2^. At the same time, the solar and lunar gravitational acceleration also have a significant effect on satellite orbit, with a magnitude both at several 1 × 10^−6^ m/s^2^. The SRP acceleration is quite stable over one day, with a magnitude at 1 × 10^−7^ m/s^2^. 

The current active GPS constellation (up to 18 April 2017) consists of 12 Block II replenishment (IIR) satellites, 7 Block II replenishment-modernized (IIR-M) satellites, and 12 Block II follow-on (IIF) satellites for a total of 31 satellites, which is under full operational capability. As we can see in Table 3, there are four different categories of satellites according to the block and satellite clock type, so four different satellites (pseudo-random noise (PRN) 2 IIR/Rubidium (Rb), PRN 5 IIR-M/Rb, PRN 3 IIF/Rb, PRN 8 IIF/Cesium (Cs)) will be used to represent all the GPS satellites in the following investigation. 

**Figure 2:**
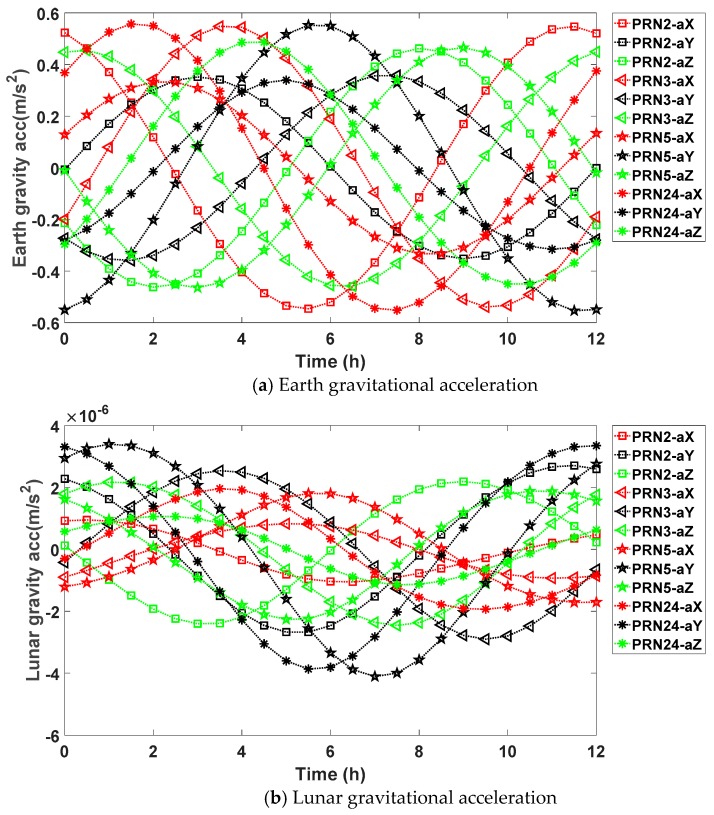

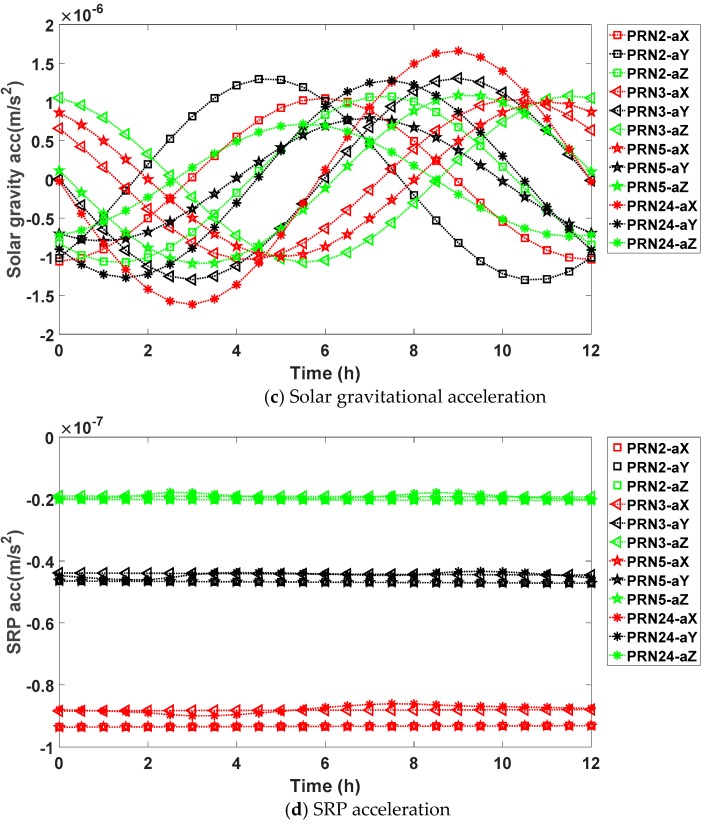
Earth gravitational acceleration, lunar gravitational acceleration, solar gravitational acceleration, and SRP acceleration over one day for GPS satellites PRN2, PRN 3, PRN 5, and PRN 24.

As we can see from the following Figure 2, the magnitudes of the accelerations are consistent with those in Table 2. The Earth’s gravitational acceleration varies between 0 to 0.6 m/s^2^, and the values for different satellite are similar. For the lunar gravitational acceleration, the absolute value peaks round 4 × 10^−6^ m/s^2^, and varies with the motion of the GPS satellite. The maximum absolute solar gravitational acceleration is around 2 × 10^−6^ m/s^2^, which is the same magnitude as the lunar gravitational acceleration. For the SRP acceleration, the magnitude is between 1 × 10^−8^ m/s^2^ and 1 × 10^−7^ m/s^2^; meanwhile, the SRP plot for PRN 2 and PRN 5 overlaps due to the small difference of SRP acceleration leveled at 1 × 10^−11^ m/s^2^.

Orbit fitting is another crucial step for generating satellite orbit IP. Firstly, setting an a priori orbit IP $x_{0}=\left[r_{3\times 1}^{0},\;v_{3\times 1}^{0},p_{9\times 1}^{0}\right]$, including three position parameters, three velocity parameters, nine SPR parameters, and the orbit error function at time $t_{k}$, will have the following form:

(2) $$\vec{v_{k}}=h(t_{k},\vec{a},x_{0})-\vec{r_{k}},\;P_{k}$$

where $\vec{v_{k}}$ is the residual, $\vec{r_{k}}$ is the observation, and $P_{k}$ is the weight matrix. After linearization by calculating the partial derivation of $h(t_{k},\vec{a},x_{0})$ with respect to $x_{0}$, Equation (2) transforms into the following Equation (3):

(3) $$\begin{array}{l} \vec{v_{k}}=H\Delta x_{0}-\vec{l},\;P_{k}\\ H=\frac{\partial h(t_{k},\vec{a},x_{0})}{\partial x_{0}}\;and\;\vec{l}=\vec{r_{k}}-h(t_{k},\vec{a},x_{0}) \end{array}$$

where H denotes the design matrix, $\Delta x_{0}$ is the IP correction, and $\vec{l}$ is the misclosure vector, while the other symbols denote the same value as above. Once Equation (3) has been constructed at all the selected epochs, then the least square can be carried out to calculate the $\Delta x_{0}$ as following:

(4) $$\Delta x_{0}={\left(\sum_{k=1}^{m}H_{k}^{T}P_{k}H_{k}\right)}^{-1}\cdot \sum_{k=1}^{m}H_{k}^{T}P_{k}l_{k}$$

Finally, the refined ${\hat{x}}_{0}$ is obtained as ${\hat{x}}_{0}=x_{0}+\Delta x_{0}$. With the estimated ${\hat{x}}_{0}$, the orbit can be predicted to the specific epoch by applying numerical integration. 

### 2.2. Generation of Satellite Orbit at User End 

The orbit numerical integration is used to find numerical approximations to the solutions of ordinary differential equations, which will be applied in the generation of high-precision orbit. A first-order differential equation is an initial value problem of the form as the following Equation (5):

(5) $$y\prime (t)=f(t,y(t)),\;y(t_{0})=y_{0}$$

Various numerical methods have been developed to solve the above equation, such as Euler’s, the Modified Euler, Midpoint, Runge–Kutta [13], and Adams–Bashforth–Moulton methods [14]. In this experiment, the Runge–Kutta and Adams–Bashforth–Moulton methods are applied. Firstly, the single step Runge–Kutta method is applied to get the start points; then, the Adams–Bashforth–Moulton multi-step method is used to predict forwards. The general expression of the Runge–Kutta method can be expressed as the following Equation (6):

(6) $$\begin{array}{l} y_{n+1}=y_{n}+h\sum_{i=1}^{s}b_{i}k_{i}\\ k_{1}=f(t_{n},y_{n})\\ k_{2}=f(t_{n}+c_{2}h,y_{n}+h(a_{21}k_{1}))\\ k_{2}=f(t_{n}+c_{3}h,y_{n}+h(a_{31}k_{1}+a_{32}k_{2}))\\ \;\vdots \\ k_{s}=f(t_{n}+c_{s}h,y_{n}+h(a_{s1}k_{1}+a_{s2}k_{2}+\cdots +a_{s,s-1}k_{s-1})) \end{array}$$

To specify a specific method, one needs to provide the integer *s* (the number of orders), and the coefficients $a_{ij}$ (for 1 ≤ *j* < *i* ≤ *s*), $b_{i}$ (for *i* = 1, 2, ... *s*) and $c_{i}$ (for *i* = 2, 3, ... *s*). If we assume that several start points have been obtained by carrying out the Runge–Kutta in a row, then we can come to the Adams–Bashforth–Moulton method, which consists of predictor (Adams–Bashforth) and corrector (Adams–Moulton) parts. Adams–Bashforth and Adams–Moulton can be expressed as the Equations (7) and (8) correspondingly:

(7) $$\begin{array}{l} y_{n}=y_{n-1}+h\sum_{s=0}^{n-1}b_{s}f(t_{s},y_{s})\\ b_{s-j-1}=\frac{{\left(-1\right)}^{j}}{j!(s-j-1)!}\int_{0}^{1}\prod_{\begin{array}{l} i=0\\ i\neq j \end{array}}^{s-1}\left(u+i\right)du,\;for\;j=0,\ldots ,s-1. \end{array}$$

(8) $$\begin{array}{l} y_{n}=y_{n-1}+h\sum_{s=0}^{n}b_{s}f(t_{s},y_{s})\\ b_{s-j}=\frac{{\left(-1\right)}^{j}}{j!(s-j)!}\int_{0}^{1}\prod_{\begin{array}{l} i=0\\ i\neq j \end{array}}^{s-1}\left(u+i-1\right)du,\;for\;j=0,\ldots ,s. \end{array}$$

**Figure 3:**
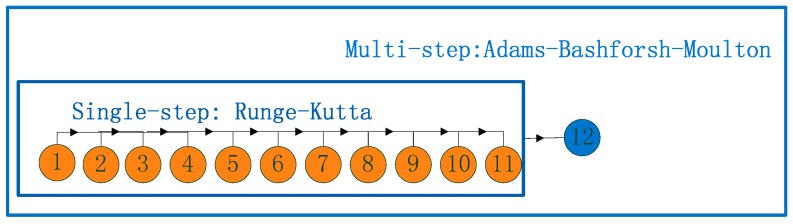
Implementation of the numerical integrator for orbit prediction.

The difference between the predictor and corrector is that the former just utilizes $f\left(t_{1},y_{1}\right)\ldots f\left(t_{n-1},y_{n-1}\right),\;y_{1}\ldots y_{n-1}$ to derive the value of $y_{n}$, while the latter also makes use of $f\left(t_{n},y_{n}\right),y_{n}$ to get $y_{n}$. When the multi-step predictor and corrector are implemented together, the accuracy will be improved. The implementation of the integrator is presented in Figure 3:

In terms of orbit generation, the y can be set as the (r,V) where r and V are satellite position and velocity in three different directions. Then, $f\left(t_{s},y_{s}\right)=\left(V,\alpha \right)$, where α is the acceleration in three different directions. If $y_{0}$ and the expression of $f\left(t_{s},y_{s}\right)$ are known, then the orbit prediction can be carried out forwards or backwards. Once the orbit IP, including position $r_{3\times 1}^{0}$, velocity $v_{3\times 1}^{0}$, SRP vector $p_{9\times 1}^{0}$ and $ERP_{6\times 1}^{0}$ are known, the satellite orbit at time $t_{k}$ can be generated with the abovementioned numeric integrator, which can also be expressed as a function of all the related parameters:

(9) $${\tilde{r}}_{k}=h(t_{k},\vec{a},r_{3\times 1}^{0},v_{3\times 1}^{0},p_{9\times 1}^{0})$$

## 3. Comparison with the IGS Final Products 

**Figure 4:**
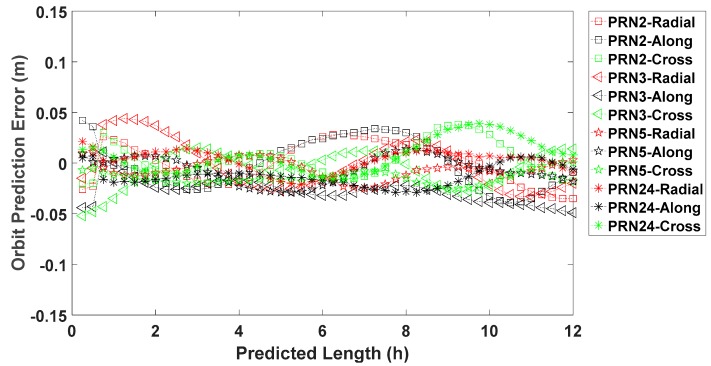
Orbit prediction error in the radial, along-track and cross-track directions over 12 h on DOY 109 of 2017 for GPS satellite PRN 2, PRN 3, PRN 5 and PRN 24.

The real-time satellite orbits from day of year (DOY) 105 to 111 of 2017 are obtained from IGS RTS through the Internet. The stream used in the experiment is the IGS 01 with a 5 s update rate for satellite orbit (encoded in message type 1060). Meanwhile, the IGS final products from DOY 106 to 112 of 2017 are downloaded through ftp as the reference. Additionally, the satellite reference center for IGS 01 is transferred from the antenna phase center to the satellite center of mass before the prediction, with the phase center offset in the IGS antenna files. In this part, satellite PRN2, PRN 3, PRN 5, and PRN 24 representing different kinds of GPS satellites, are selected again to do the orbit prediction on DOY 109 of 2017. As we can see from Figure 4, the orbit prediction error over 12 h is smaller than 5 cm for all the four satellites in the radial, along-track, and cross-track directions. The average RMSE of the predicted orbit over 12 h for all four satellites are 0.027 m, 0.024 m, and 0.032 m for the radial, along-track and cross track directions, respectively.

**Figure 5:**
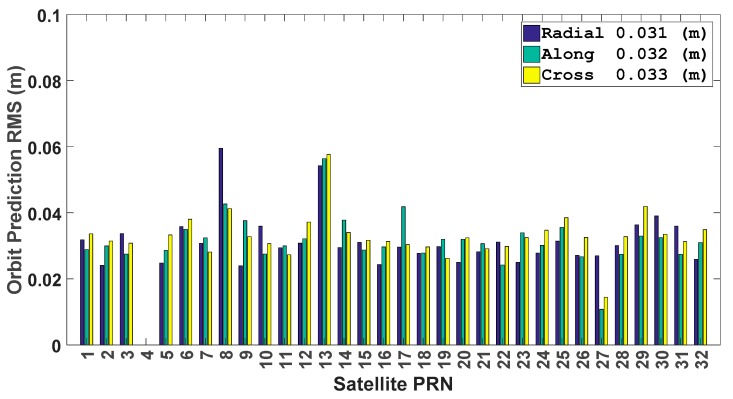
Average orbit prediction RMSE over 12 h of all GPS satellites from DOY 106 to 112 of 2017.

Afterwards, the GPS satellite orbit prediction is carried out from DOY 106 to 112 of 2017. For each day, the satellite orbit is predicted for 12 h, and then the average RMSE values for the whole week are calculated. The average RMSE of the orbit prediction is shown in following Figure 5. As we can see, the average RMSE values in all three directions are smaller than 0.060 m for all GPS satellites, and the average RMSE in three directions are close to each other. Meanwhile, the average RMSE values of the 31 satellites are 0.031, 0.032, and 0.033 m for the radial, along-track and cross-track directions.

**Figure 6:**
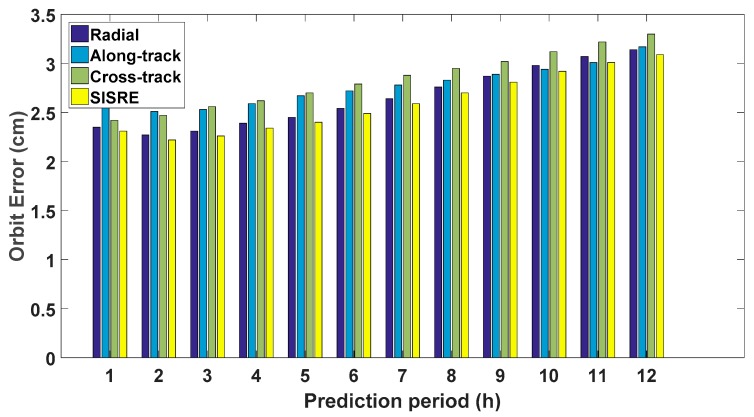
Average RMSE of the predicted GPS satellite orbit over 12 h.

To investigate the effects of the orbit on positioning, the Signal In Space Range Error (SISRE (orb)) [15,16] are calculated according to the following equation.

(10) $$\mathrm{SISRE}(\mathrm{orb})=\sqrt{w_{R}^{2}\cdot R^{2}+w_{A,C}^{2}\left(A^{2}+C^{2}\right)}$$

where weight factors $w_{R}$ is the statistical constribution of radial (*R*), $w_{A,C}^{2}$ is the statistical constribution of along-track (*A*) and cross-track (*C*) errors to the line-of-sight ranging error. For GPS, $w_{R}$ and $w_{A,C}^{2}$ are 0.98 and 1/49, respectively [16]. The average RMSE of all satellites and SISRE over different periods, namely from 1 h to 12 h, are plotted in the following Figure 6.

The main trend of the RMSE of the predicted orbit is increasing gradually with the prediction periods. The average RMSE are 2.35, 2.58, and 2.42 cm for the predicted orbit over 1 h in radial, along-track, and cross-track directions, respectively. For predictions over 6 h, the average RMSE just increses slightly to 2.54, 2.72, and 2.79 cm in each of the three directions. Even for predictions over 12 h, the average RMSE is 3.14, 3.17, and 3.30 cm. For the SISRE(orb), the value increases from 2.31 to 3.09 cm with the prediction periods. We can see that the predicted orbit is accurate and more investigation is carried out with PPP.

## 4. PPP Settings and Experiments

In order to investigate the accuracy of the predicted orbit deeply, the experiments in positioning domain are carried out, including the static PPP and kinematic PPP (vehicle based).

### 4.1. Static PPP

**Figure 7:**
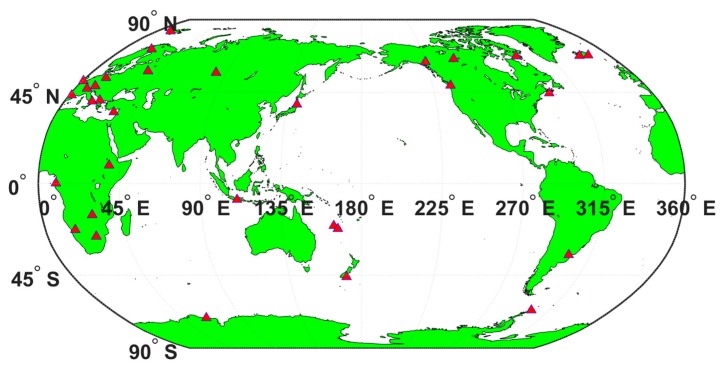
The distribution of the selected IGS stations for static PPP tests.

For the static PPP, the GPS observation data from 32 globally distributed stations are downloaded through ftp on DOY 108 of 2017, which is shown in Figure 7. 

The static PPP experiment is carried out on 18th April 2017 for 12 h and the predicted satellite orbit and the IGS final clock products with 30 s sample rate are used. In this case, the satellite orbit and clock are inconsistent since the satellite clock products are not calculated together with—or based on—the predicted satellite orbit. However, this is the case even for real-time PPP with IGS RTS due to the latency issue. The satellite orbit and clock are consistent at the reference time for the satellite clock is real-time estimated based on the orbit. However, both the satellite orbit and clock need to be predicted to the real-time observation epoch, then the consistency between satellite orbit and clock are broken after this process. The IGS final clock products are accurate and don’t compensate for the predicted satellite orbit when used together, so the predicted orbit error can be more directly presented in the PPP.

The basic filter settings for the PPP are listed in the following table, and the station coordinates are estimated along with the receiver clock, tropospheric zenith wet delay, and ambiguity parameters. The details setting of the PPP filter is listed in Table 4. 

**Figure 8:**
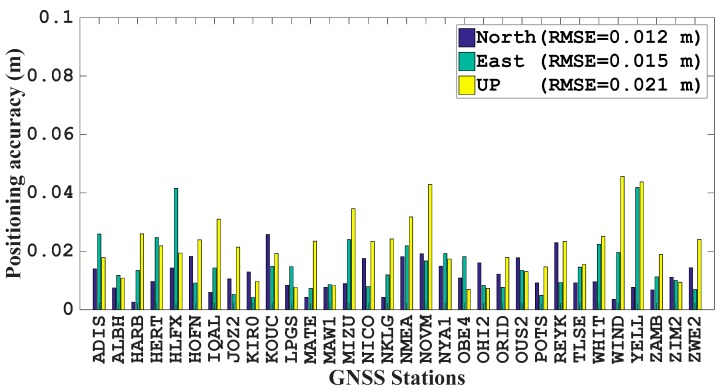
Positioning accuracy of 32 globally distributed IGS stations on 18 April 2017 with the predicted GPS orbit.

The reference coordinates for all of the IGS stations are obtained from the Scripps Orbit and Permanent Array Center [17]. Then, the static PPP accuracy is shown in Figure 8. As we can see from Figure 8, the RMSE values for all of the selected stations are less than 5 cm in all three directions. The various positioning accuracy is due to the different visibility of satellites as well as the geometry. For stations like MAW1 station, the positioning RMSE values are around 1 cm for all three directions, while the vertical positioning RMSE of WIND station can reach around 4 cm. When we take all the stations into average, the positioning RMSE values are 0.012, 0.015, and 0.021 m for the north, east and vertical directions, respectively. 

### 4.2. Kinematic PPP

**Figure 9:**
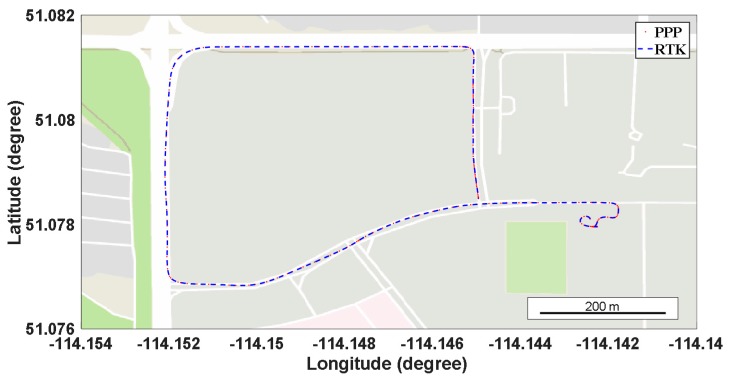
Trajectory of the kinematic PPP testing around the University of Calgary, Canada.

For the kinematic PPP, the field test is carried out around the children’s hospital in Calgary, Canada with a Trimble R10 receiver. The sample rate of the GNSS observation is 1 s. Meanwhile, the IGS station (UCAL) located 3 km away from the rover is used as the base station for real-time kinematic (RTK). The field test is operated from 20:23:00 to 23:00:00 (UTC time) on 18 April 2017. During the field test, the speed limit for the road section is 50 km/h, and several red lights are experienced at the intersections. The following Figure 9 shows the trajectory of the field test for kinematic PPP, in which the red dots represent the PPP solutions, while the blue dash lines denote the RTK results. 

**Figure 10:**
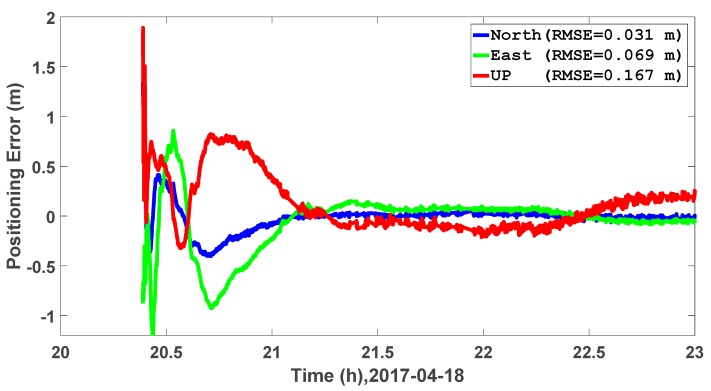
Kinematic PPP results around a children’s hospital in Calgary on 18 April 2017.

As we can see from Figure 9, the PPP and RTK solutions coincide with each other very well, and more detailed comparison is given in Figure 10. The kinematic PPP takes around 40 min to converge to 0.5 m, while the predicted orbit and RMSE of the kinematic PPP after convergence is 0.031, 0.069, and 0.167 m in the north, east, and vertical directions, respectively. 

## 5. Conclusions 

This paper firstly introduces a new real-time PPP system in terms of server end, communication end, and user end; then, the orbit prediction is discussed, including the generation of a low update rate IP at the sever end, and orbit predictions of satellite orbit at the user end. Through the designed experiment, with satellite PRN2, PRN 3, PRN 5, and PRN 24 representing all the GPS satellites, we can confirm the magnitude of different accelerations, and that the Earth’s gravitational acceleration is 1 × 10^−1^ m/s^2^. For the lunar and solar gravitational acceleration, both are at 1 × 10^−6^ m/s^2^. For the SRP acceleration, the magnitude is between 1 × 10^−8^ m/s^2^ and 1 × 10^−7^ m/s^2^. When comparing the predicted orbit with the IGS final orbit products over different prediction lengths, namely from 1 h to 12 h, the orbit RMSE increases slightly with the increase of the prediction length, and the average RMSE of the 31 satellites are 0.031, 0.032, and 0.033 m for radial, along-track, and cross-track directions over 12 h. Further positioning experiments show that the predicted orbit is accurate enough for both static and kinematic PPP. For static PPP with globally distributed IGS stations, the average positioning RMSE values are 0.012, 0.015, and 0.021 m for the north, east and vertical directions. When comes to the kinematic PPP, the average positioning RMSE values are 0.031, 0.069, and 0.167 m in the, east, and vertical directions, respectively.

## Figures and Tables

**Table 1:** Satellite orbit IP in the new real-time PPP system.

	Specific Parameters	Parameter Numbers
Satellite orbit IP	Satellite coordinates	(3*n*)
	Satellite velocities	(3*n*)
	Satellite SRP parameters	(5*n* or 9*n*)

Notes: *n* is the number of satellites.

**Table 2:** Effects of perturbing forces on global positioning system (GPS) satellites and the models applied [12].

Items	Models	Acceleration (m/s^2^)
Earth gravitation	EGM2008 with degree 12 and order 12 with tide models	1 × 10^−1^
Solar gravitation	The law of gravity and solar position from DE405 ephemeris	1 × 10^−6^
Lunar gravitation	The law of gravity and lunar position from DE405 ephemeris	1 × 10^−6^
SRP	ECOM with 9 parameters	1 × 10^−7^
Numerical integrator	7/8 order single-step Runge-Kutta (75 s)	Not applicable
	12 order multi-steps Adams-Bashforsh-Moulton (900 s)	Not applicable
Arc length	24 h	Not applicable
Sample rate	900 s	Not applicable

**Table 3:** Block type, PRN and clock type of GPS (up to 18 April 2017).

	Satellite Space Vehicle Numbers (SVN)/PRN
IIR/Rb	43/13, 46/11, 51/20, 44/28, 41/14, 54/18, 56/16, 45/21, 47/22, 59/19, 60/23, 61/2
IIR-M/Rb	53/17, 52/31, 58/12, 55/15, 57/29, 48/07, 50/5
IIF/Rb	62/25, 63/1, 66/27, 64/30, 67/6, 68/9, 69/3, 71/26, 73/10, 70/32
IIF/Cs	65/24, 72/08

**Table 4:** Filter settings for the PPP.

	Settings
Constellation	GPS satellites
Combination mode	Ionosphere-free phase and code combinations
Signal selection	GPS L1/L2
Sampling rate	1 s
Elevation mask	7°
Observation weight	Elevation dependent weight
Tropospheric zenith hydrostatic delay	GPT model
Tropospheric zenith wet delay	Initial model + estimated (random walk process)
Troposphere mapping function	GMF
Phase wind-up	Corrected
Sagnac effect, relativistic effect	IS-GPS-200
Satellite/receiver phase center correction	Corrected with IGS absolute correction model
Solid tides effect	IERS conventions [9]
Receive clock	Estimated, white noise
Station coordinates	Estimated

## Abbreviations

**Acronyms**	**Full Name**
CODE	Center for Orbit Determination in Europe
Cs	Ceasium
DE	Development Ephemeris
DOY	Day Of Year
ECOM	Empirical CODE Orbit Model
EGM	Earth Gravitational Model
GNSS	Global Navigation Satellite System
IGS	International GNSS Service
IIF	II Follow-on
IIR	II Replenishment
IIR-M	II Replenishment-Modernized
IP	Initial Parameters
JPL	Jet Propulsion Laboratory
PPP	Precise point positioning
PRN	Pseudo Random Noise
Rb	Rubidium
RMSE	Root Mean Square Error
RTK	Real Time Kinematic
RTS	Real-Time Service
SVN	Space Vehicle numbers
SRP	Solar Radiation Pressure
ZWD	Zenith Wet Delay
